# Interdisciplinary Public Health Reasoning and Epidemic Modelling: The Case of Black Death

**DOI:** 10.3201/eid1202.051330

**Published:** 2006-02

**Authors:** Martin I. Meltzer

**Affiliations:** * Centers for Disease Control and Prevention, Atlanta, Georgia, USA

**Keywords:** epidemic modeling, mathematical modeling, black death, plague, book review

Because public health officials increasingly rely on mathematical models to help them prevent and control diseases, this book is a very timely addition to the literature. The authors' overall theme is that generating accurate and useful (to public health officials) mathematical models of disease epidemiology and the impact of interventions requires a true interdisciplinary approach. They maintain that there is a need to incorporate knowledge and data from both physical and life sciences into such models. For example, the authors argue that information should be included on the clinical (life science) aspects of a disease (e.g., incubation period, efficiency of transmission), as well as on how the disease spreads geographically (physical science) over time (different communities could experience very different patterns of spread). They also note that the onus of improving models does not lie solely with the modelers. Users, particularly public health officials, are part of an interdisciplinary team. Consequently, users have to better acquaint themselves with what models can and cannot do (i.e., the production of mathematical "black boxes" is not entirely the fault of the modelers). The authors illustrate their themes by comprehensively examining the spread of the Black Death in the mid-1300s.

Many Emerging Infectious Diseases readers are likely to find this book overly technical, containing many mathematical formulas, mathematical notations, and complex graphs. However, a reader willing to ignore the potential intimidation of such material may find interesting discussions of modeling philosophy, such as the importance of including probability (i.e., uncertainty or "randomness") and the impact of space-time. For the latter, even the most ardently nonmathematical reader is likely to be fascinated by the maps in Chapter 5 that depict the spread of the Black Death. The data required to model (map out) the spread of disease over time and space require intensive "detective work," to which epidemiologists and public health officials can readily contribute. Readers interested in the background data related to the epidemiology of the Black Death will probably enjoy perusing the detailed, annotated data appendices.

This would be a fine addition to a technical library as a resource for persons who conduct sophisticated mathematical modeling. However, persons looking for a more general historic overview of the Black Death (how it spread and its consequences) would be advised to consider other works such as those by McNeill (*1*) or Cantor (*2*).
